# Can Variability of Pattern ERG Signal Help to Detect Retinal Ganglion Cells Dysfunction in Glaucomatous Eyes?

**DOI:** 10.1155/2015/571314

**Published:** 2015-06-08

**Authors:** Alberto Mavilio, Francesca Scrimieri, Donato Errico

**Affiliations:** ^1^Social Health District, Glaucoma Center, Azienda Sanitaria Locale, Via Fermi, 72015 Fasano, Brindisi, Italy; ^2^Ophthalmology Unit, Glaucoma Service, Azienda Ospedaliera “Cardinale G. Panico”, 73039 Tricase, Lecce, Italy

## Abstract

*Objective*. To evaluate variability of steady-state pattern electroretinogram (SS-PERG) signal in normal, suspected, and glaucomatous eyes. *Methods*. Twenty-one subjects with suspected glaucoma due to disc abnormalities (GS), 37 patients with early glaucoma (EG), and 24 normal control (NC) were tested with spectral-domain optical coherence tomography (SD-OCT), standard automated perimetry (SAP), and SS-PERG. Mean deviation (MD), pattern standard deviation (PSD), retinal nerve fiber layer (RNFL), and ganglionar complex cells (GCC) were evaluated. The SS-PERG was recorded five consecutive times and the amplitude and phase of second harmonic were measured. PERG amplitude and coefficient of variation of phase (CVphase) were recorded, and correlation with structural and functional parameters of disease, by means of one-way ANOVA and Pearson's correlation, was analysed. *Results*. PERG amplitude was reduced, as expression of retinal ganglion cells (RGCs) dysfunction, in EG patients and GS subjects compared to NC patients (*P* < 0.0001). CVphase was significantly increased in EG patients and GS subjects, compared to healthy (*P* < 0.0001), and it was also correlated with PSD (*P* = 0.0009), GCC (*P* = 0.028), and RNFL (*P* = 0.0078) only in EG patients. *Conclusions*. Increased intrasession variability of phase in suspected glaucomatous eyes may be a sign of RGCs dysfunction.

## 1. Introduction

Glaucoma is a progressive optic neuropathy characterized by death of retinal ganglion cells (RGCs), clinically manifested as typical alterations of the optic nerve head (ONH) and retinal nerve fiber layer (RNFL) correlated with visual field defects.

The standard automated perimetry (SAP) is the main tool for the detection of visual field loss. However, the subjective nature of the test and the fact that the examination reveals glaucomatous defects only when 30 to 40% of the fibers have already been lost [1, 2] have increased the interest of research towards alternative diagnostic tools.

The spectral-domain optical coherence tomography (SD-OCT), a good surrogate accepted for the diagnosis of glaucoma, has been shown to objectively measure ONH and RNFL [3–5].

Because most of the retinal ganglion cells are located in the macula, the study of this area, in particular the ganglion cells complex (GCC), has been proposed in the early evaluation of glaucoma variations, in addition to the changes that occur in ONH and RNFL [6–10].

Pattern electroretinogram (PERG) alterations reflect the electrical activity of RGCs [11, 12] and has been widely used to detect the loss of function of RGCs in glaucoma [13, 14]. Cross-sectional studies have shown that PERG is frequently altered in glaucoma suspects (GS) and patients with early glaucoma with respect to normal controls [13–19].

PERG has been shown to be abnormal before both the occurrence of visual field defects, as measured by SAP, and RNFL loss, as assessed by OCT [13].

An optimized model of PERG for glaucoma screening (PERGLA) is a relatively new diagnostic tool and fast and user-friendly for the evaluation RGCs dysfunction [20, 21].

The steady-state PERG is recorded in response to stimulus of high temporal frequency [22]. Better than a transient stimulus (slow), a steady-state stimulus (fast) is able to show a glaucomatous dysfunction since it submits the RGCs to a greater metabolic stress [20]. The steady-state stimulus determines a sinusoidal response that is analysed by the Fourier transform [23–25]. In this way, the second harmonic, that is, the harmonic that has a frequency twice that of the stimulus, is isolated. Two components of this harmonic, that is, amplitude and phase, show typical alterations in glaucoma. In particular, the amplitude is reduced in patients with glaucoma and ocular high pressure (OHT) compared to healthy subjects [14, 21], while the phase remains constant or at most tends to delay with age [20]. Steady-state PERG has been reported to have high test-retest repeatability [26]. However it is very important to work with a good signal-to-noise ratio (SNR) [27]. In particular the phase variability has been showed to be very limited in the retest within and between trials [16, 26].

The biological variability of a measurement is a physiological characteristic that can bias not only diagnostic imaging [28] but also the different adaptation of the bioelectrical response to an external visual stimulus [29, 30].

Variations of the phase are little affected by opacity of the media and deterioration of optics that may cause a nonspecific reduction of PERG amplitude [31].

If the variability of the phase is the expression of a dysfunction of RGCs that precedes cell death, we hypothesized that the within-trial variability of the PERG signal, individual test-retest of the same eye of early glaucoma patients, is greater than the one physiologically present in healthy individuals. Therefore, we checked if such variability correlates with markers of disease severity such as retinal thickness and visual field indices.

## 2. Materials and Methods

Participants were recruited from the Glaucoma Center of the Brindisi Social Health District, Mesagne, Italy. The participants were divided into 3 groups: early glaucoma (EG), glaucoma suspects (SG), and normal control (NC).

A total of eighty-two eyes were included. The criteria for classification in the EG group, in accordance with the EGS guidelines (http://www.eugs.org/eng/EGS_guidelines.asp), were as follows: appearance of the optic disc and peripapillary nerve fiber layer suspected for glaucoma damage (increased ratio cup/disc, asymmetry ratio of cup/disc, notch or narrowing of the neuroretinal rim, disc haemorrhage, and thinning of the peripapillary nerve fiber layer) or visual field suspicious for glaucomatous damage in the absence of clinical signs of other optic neuropathies (nasal step, paracentral scotoma, and altitudinal defect) or a constant elevated intraocular pressure.

The severity of glaucoma was evaluated functionally by means of SAP and anatomically by means of RNFL and GCC thickness measurement by SD-OCT.

Thirty-seven patients had early glaucoma (EG), defined as consecutive repeatable abnormal SAP results according to the normative database of the instrument; 21 had suspected glaucoma, defined as optical discs apparently abnormal (presence of thinning of the neuroretinal rim or localized or diffuse RNFL defects indicative of glaucoma as evaluated by Stereo photography of the fundus) without repeatable abnormal SAP results. 24 age-matched healthy subjects, defined as those with IOP <22 mmHg with no history of elevated IOP, optic disc apparently healthy, and normal SAP repeatable results, were included in the control group. The EG patients were under medical topical treatment with beta-blockers, prostaglandin analogues, and alpha adrenergic receptors eye drops. Each participant of the trial underwent a comprehensive ophthalmic evaluation, including review of medical history, best-corrected visual acuity testing, IOP measuring by means of Goldmann applanation tonometry, ultrasound pachymetry (Pachmate GH55), slit lamp biomicroscopy, gonioscopy, and dilated fundus examination with a 78 D lens. All participants had best-corrected visual acuity ≥20/30 (Snellen), spherical refraction within ±5.0 D and cylinder correction within ±2.0 D, transparent ocular media (nuclear color/opalescence, cortical or posterior subcapsular lens opacity <1) according to the Lens Opacity Classification System III, and open angle on gonioscopy. Coexisting retinal disease, diabetes, Parkinson's disease, or nonglaucomatous optic neuropathy, potentially able to determine nonspecific PERG abnormality, were excluded [32–34].

One eye per patient who met the criteria mentioned above was included in the study. When both eyes of the patient were eligible, the one with best-corrected visual acuity was selected. In case of equal visual acuity, right eye, by convention, was selected for evaluation.

This trial followed the tenets of the Declaration of Helsinki for human studies. The study was approved by Ethical Committee of the Brindisi Social Health District. Informed written consent was obtained by all subjects after the nature of the test and possible risks were explained in detail.

### 2.1. Spectral-Domain Optical Coherence Tomography

Peripapillary RNFL thickness was assessed by Zeiss Cirrus HD-OCT 500 (software version 7.0.1.290, Carl Zeiss Meditec, Dublin, CA). The protocol Optic Disc Cube 200 × 200 was used to perform a circular scan 3.46 mm in diameter, which was automatically targeted around the optic disc to provide the RNFL thickness of the four quadrants and each of the 12 clock-hour positions. The protocol Macular Cube 512 × 128 was used to obtain measurements of retinal ganglion cell macular thickness.

All images were obtained by the same experienced technician with a quality score of at least 7/10. Three consecutive scans of the optic disc and macular region were acquired and analysed for each eye. Measurements of RNFL and GCC were averaged using the data of each of the three scans.

### 2.2. Standard Automated Perimetry

The visual field was assessed by means of a Humphrey Field Analyzer, model 745i II (Carl Zeiss Meditec, Germany), using the 24-2 test program, SITA standard strategy. Near addition was added to the refractive correction, where needed. If fixation losses were greater than 20% and false-positive or false-negative results were higher than 15%, the test was repeated. At least 2 reliable SAPs were performed to minimize the learning effect [35]. Visual field defects were defined as being typically glaucomatous when a standard deviation of the model (PSD) significantly higher than the 5% level and/or a glaucoma hemifield test outside normal limits was recorded.

### 2.3. Pattern Electroretinogram

PERG was recorded by means of an instrument supplied by our laboratory (RETIMAX Advanced version 4.3 CSO, Pisa, Italy), using a method similar to the paradigm PERGLA [20], with some minor changes made by our laboratory.

We used as a stimulus horizontal bars with a spatial frequency of 1.7 cycles/degree, which resulted from previous studies as the most sensitive in detecting RGCs dysfunction in early glaucoma [36, 37], modulated in counterphase at 15 reversals/second and electronically generated on a high-resolution LCD monitor (contrast: 90%; luminance: 80 cd/m^2^; field size: 24° [width] × 24° [height]). The subjects had undilated pupils, of size between 3 and 4 mm, with an appropriate correction for the working distance (57 cm). The signals were recorded from a skin electrode 9 mm Ag/AgCl placed on the lower eyelid. A similar electrode, placed on the lid of the not stimulated eye, was used as a reference, as described in other studies [38].

**Figure 1:**
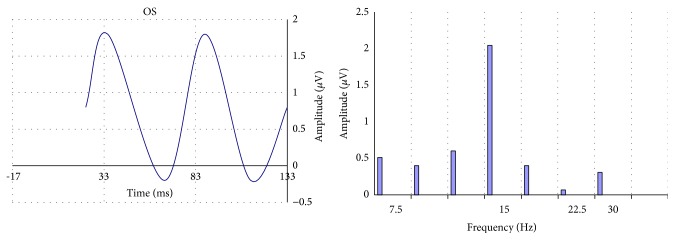
Example of steady-state PERGs of left eyes, presented in time domain (line chart) and frequency domain (bar chart). At a frequency of the stimulus of 7.5 Hz the second harmonic is observed at 15 Hz.

**Figure 2:**
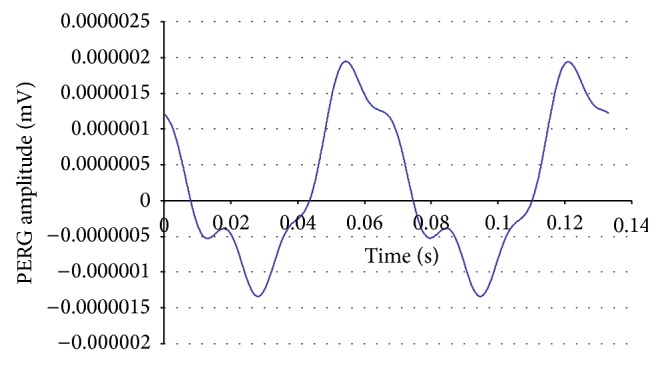
Example of steady-state PERGs recorded in response to a pattern of horizontal gratings (1.7 cy/deg. 90% contrast; 80 cd/m^2^ mean luminance; field size: 24° [width] × 24° [height]) alternating at 15 times/s or every 66.6 ms.

**Figure 3:**
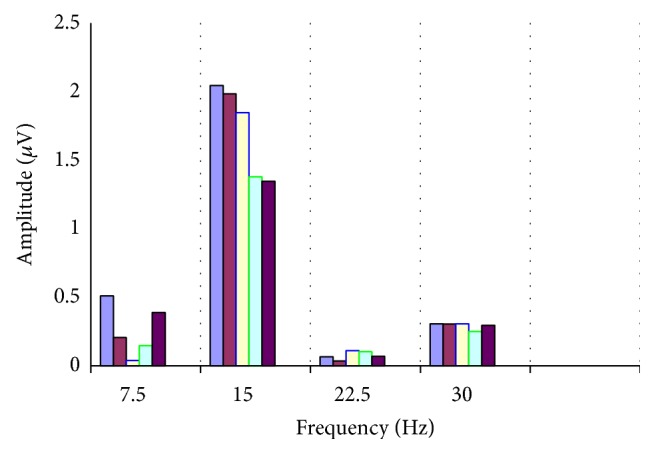
Fourier spectrum of 5 consecutive tests of steady-state PERG in the same subject. The bar chart shows the amplitude expressed in *μ*V of second harmonic of the signal in response to stimulus of 7.5 Hz.

In all cases the impedance was below 5 k. The responses were amplified (gain of 100.000), filtered (bandwidth: 130 Hz), and sampled with a resolution of 12 bits. The analysis time was 133 ms, equal to the time of presentation of the stimulus (Figure 1). An average (100 events), with automatic rejection of artefacts, was obtained. Five consecutive tests were recorded with a short break, so the duration of the examination was no more than 5 minutes per eye (the total duration being no longer than a visual field examination). The data were then exported to a text file. The amplitude (*μ*V) and phase (*π* rad) of the second harmonic were then analysed with the Fourier transform (Figures 2 and 3) using a special software programmed by one of the authors (Alberto Mavilio).

The repeatability of the amplitude and phase of the second harmonic was calculated as coefficients of variation (CV, the ratio of the measurement standard deviation to the mean), CVamp (coefficient of variation of amplitude), and CVphase (coefficient of variation of phase), respectively, and as the intraclass correlation coefficients (ICC, describing proportion of total variance accounted for by within-subject variation).

The noise level obtained by recording a response to an occluded stimulus was ≤0.097 ± 0 : 04 *μ*V in both normal subjects and patients.

In our laboratory the phase decreases with increasing peak time; that is, a delayed response corresponds to lower phase values.

At a reversal rate of 15 Hz the modulo value of 2 *π* rad corresponds to 66.6 ms (1/15 ∗ 1000 = 66.6 ms). As described previously [31], to avoid the inherent discontinuity of phase, the recorded value was subtracted from the value of the modulo (2 less than the recorded value). This is needed to prevent negative values of the phase that may affect the calculation of the coefficient of variation.

Statistical analyses were performed using a commercially available software (MedCalc 13.3.1.0). A *P* value of ≤ 0.05 was considered statistically significant.

## 3. Results

Demographic, structural, and functional data are shown in Tables 1, 2, and 3.

The differences of the variables between groups were analysed using one-way ANOVA analysis of variance, with Bonferroni adjustment. Both linear and logarithmic regressions were used for structural measurements; RNFL and GCC were expressed in linear units (micrometers); functional measurements, PSD, and MD were expressed in logarithmic units (decibels). Pearson correlation in PERG amplitude, phase, coefficients of variation, thickness measurements SD-OCT, and visual field indices are shown in Table 4.

Age, which can affect the amplitude of the PERG and retinal thickness, was not a confounding factor; in fact there was no statistically significant difference of age between all groups (see Table 1).

Because the RNFL thickness shows a slight decrease with age, as suggested by previous studies [39, 40], the data were adjusted for age by a factor of 0.2 *μ*m/year (0.18%/year).

The IOP values were significantly higher in the GS group (17.95 ± 1.65 mmHg) than in the EG group (15.67 ± 1.13 mmHg), because the EG patients were in drug treatment.

PERG amplitude was reduced in EG (0.96 ± 0.33 *μ*V) and GS (0.96 ± 0.27 *μ*V) subjects with respect to NC (1.20 ± 0.26 *μ*V, *P* < 0.0001) and was weakly associated with RNFL thickness (*r* = 0.444, *P* = 0.0059).

CVamp correlates negatively with GCC (*r* = −0.379, *P* = 0.0206), while CVphase correlates better with RNFL (*r* = 0.427, *P* = 0.0083) than with CGG (*r* = 0.361, *P* = 0.0283).

Finally, CVphase correlates fairly strongly with PSD (*r* = −0.524, *P* < 0.0009) and weakly with IOP (*r* = 0.362, *P* = 0.0277).

When the data from normal eyes were entered into a multiple linear regression analysis, with CVphase and CVamp as independent variables and PSD as dependent variable, the significance of the coefficient of variation for CVphase term and for CVamp term was not significant.

In a similar analysis of the EG group, the significance of the coefficient for the CVphase term was *P* = 0.0010 and for the CVamp term was *P* = 0.9767.  Table 5 shows the intraclass correlation coefficients (ICC).

## 4. Discussion

Our results are similar to previous studies. Many authors evaluated the PERG procedure which inspired us in this trial, the PERGLA paradigm, with regard to its reliability in the early diagnosis of glaucoma [16, 18, 19, 26, 27, 41–43].

Several studies also verified structure-function relationship using PERGLA paradigm and methods of analysis of the structure as retinal OCT [13, 28, 41, 44, 45].

Other studies reported that the total thickness of the retina is a good surrogate for glaucomatous damage ganglion cell layer measured as SD-OCT [46, 47] and that the total macular thickness was significantly associated with glaucoma [7]. Nevertheless, we observed a better association between PSD, which indicates the severity of the disease, and RNFL (*R* ^2^ = 0.1487, *P* = 0.0184) than GCC (*R* ^2^ = 0.07782, *P* = 0.0945).

Longitudinal studies have shown that the PERG amplitude is able to detect signs of glaucomatous damage before psychometric and morphological techniques [15, 48, 49].

The PERG amplitude was significantly lower in EG and GS patients (0.96 ± 0.33 *μ*V and 0.96 ± 0.27 *μ*V, resp.) compared to healthy (1.20 ± 0.26 *μ*V). Our data demonstrate that the reduction of PERG amplitude in SG subjects that, unlike EG patients, still do not show abnormal visual field and reduced retinal thickness, is an indicator of RGCs dysfunction. As noted by other authors [13] PERG amplitude is weakly but significantly associated with RNFL thickness reduction in EG patients (*R* ^2^ = 0.1975, *P* = 0.0059), whereas PERG phase in the current study did not show statistically significant differences between the subjects of the different groups.

It must be said that our trial PERG amplitude was higher than that previously reported by Bowd et al. [18] (0.96 *μ*V compared to 0.83 *μ*V) and similar to that previously reported by in healthy eyes (1.2 *μ*V compared to 1.1 *μ*V [20]). This can be explained by the different degree of severity of glaucoma, worse in Bowd patients (MD −9.0 dB), compared to −3.0 dB found in our EG patients.

Amplitude in the first test tended to be greater than that in successive tests. This finding may be related to the percentage of decrease in the amplitude due to adaptation to the stimulus PERG [30]. For this reason we have focused our attention on CVphase.

It is known that amplitude and phase of the PERG represent two distinct aspects of neural activity [31]. Briefly, the amplitude is related to the number of neurons; the phase delay is a further indicator of viability of activated neurons that may or may not be associated with amplitude reduction and in particular may mean that RGCs active respond more slowly. The last becomes progressively delayed with aging [20] and may be further delayed in early glaucoma [21].

Falsini et al. [44] showed that the loss of RGCs electrophysiological function is relatively greater than expected from the anatomical loss of RGCs axons in early glaucoma and that RNFL thickness reduction, less than expected, could be explained by glial remodelling.

In addition, changes in dendrites typically precede neuronal loss and lead to a reduction in the responsiveness of RGCs in glaucoma [50].

PERG generation includes a mixed population of RGCs [51], which are commonly divided into two major classes: P-cells (approximately 80% of the total) and M-cells (approximately 10%). M-cells are much more sensitive to changes than P-cells [52], and their response is temporally faster than P-cells [53], the first responding promptly to the steady-state PERG stimulus.

Since M-cells are relatively few and sparse, even a partial malfunction can be early detected by doubling perimetry frequency [54]. The increase of CVphase in SG subjects suggests that in early glaucoma there would be a progressive loss of the ability of RGCs to adapt in response to the growing demand for energy associated with the high-contrast PERG stimulus [55].

Porciatti and Ventura reported that PERG phase delays suggest pathophysiological mechanisms such as dendritic dysfunction or delay in axonal transport and may represent an opportunity to detect RGCs dysfunction preceding cell death [30].

In this study we demonstrated a significant but modest relationship between the RGCs function using steady-state PERG and some structural measures such as GCC and RNFL measured by OCT (see Table 4). These results suggest that a population of viable RGCs responds with reduced activity, thus signalling a period of discomfort that precedes cell death.

The primary purpose of this study was to evaluate the variability of the PERG signal because, in our opinion, it is increased more in patients suffering from glaucoma than in healthy subjects. In glaucoma suspects, reduced amplitude and increased variability of the phase of the PERG may indicate the presence of a functional impairment.

In fact, the variability of PERG signal has been evaluated in several studies: Bowd et al. [16] studied medium amplitudes and phases, their noise level, SNR, within-subject variability, CV, and ICC PERGLA recordings for within and between trials.

For amplitude, Bowd et al. observed that the variability of successive measurements was approximately 10% to 12% for healthy eyes and glaucoma patients, respectively. These values were similar within and between trials.

As for the phase, repeat measurement variability observed in this study was very low, about 1% to 2%; furthermore, ICC indicated that the percentage of total variance accounted for by intrasubject variation was similar in within and between trials and for healthy eyes and patient (range: 82% to 92%).

We observed within-trial variability of the amplitude higher than previous study, on the order of 15–20% for glaucoma patients and 10% for controls. The variability of the phase was also higher than quoted study, of 8–10% for glaucomatous eyes versus 3-4% for healthy subjects.

In this study the variability of the phase has been observed not as an element of repeatability of the method but as a discriminating feature between healthy and diseased eyes. This approach can help to detect nonspecific reduction in the amplitude of PERG.

In fact, the phase is not delayed when the contrast stimulus is artificially deteriorated by simulating the visual acuity reduction due to the cataract [30]; that is, conditions like cataract reduce the PERG amplitude but do not influence the phase delay. In other words, while the PERG amplitude and its variability may depend on the opacity of the media, the phase delay and its variability, as showed in our work, are not affected by the same conditions and may express RGCs dysfunction.

In our study we did not evaluate phase delay, but its greater variability that may be a sign of RGCs dysfunction.

**Figure 4:**
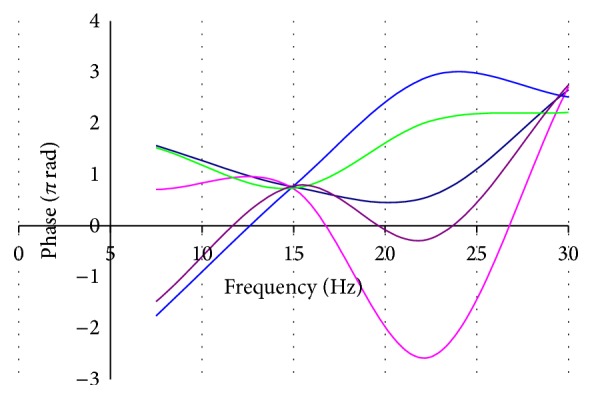
Five consecutive tests of steady-state PERG in the same subject. The line chart shows the trend of the phase in the frequency domain from 7.5 Hz to 30 Hz. The phase always passes from the same point at 15 Hz that corresponds to the second harmonic of the signal in response to stimulus of 7.5 Hz.

If you look at the phase in the frequency domain (Figure 4), by repeating the test you will see that it has an almost chaotic behavior but passes always from the same point that corresponds with the frequency of the second harmonic.

CVamp and CVphase were significantly lower in healthy individuals (3.54 ± 1.13%), compared to the groups GS (7.30 ± 2.51%) and EG (8.97 ± 2.52%), respectively (see Table 1).

Our work did not include between-proof recordings, but Fredette et al. [26] observed the test-retest variability of the PERG amplitude, expressed in terms of standard deviation (SD) of the results obtained for each subject of glaucoma during the 5 sessions in 5 different days. They also assessed the intrinsic variability (intratest), which was defined as the standard deviation of 2 consecutive recordings divided by the square root of 2. Fredette et al. argue that the amplitude variability could not be used to discriminate healthy from the glaucomatous eyes because they attributed the greater variability amplitude to the reduced amplitude of the signal in glaucomatous patients. In fact, mean amplitude and variability in their work were correlated significantly (*r* ^2^ = 0.164, *P* = 0.003), indicating that the variability amplitude was due to the low SNR.

Our trial showed no significant association between amplitude and SD (*r* ^2^ = 0.010, *P* = 0.546), probably because the amplitude of our signal and probably also our SNR were higher than measurement previously reported by Fredette et al. (0.09 ± 0 : 04 *μ*V versus 0.08 ± 0.03 *μ*V).

In addition, our trial showed no association between PERG amplitude and CVphase (*r* ^2^ = 0.001, *P* = 0.821), confirming that variability of the phase was not dependent on the magnitude of the signal.

The structure-function relationship between CVphase and RNFL or GCC was significant only in EG group (Table 4).

The intraclass correlation coefficients indicated that the percentage of the total variance represented by the within-trial variation (i.e., the measurement reliability) was very low with regard to both the phase (0.9124 to 0.9692 95% CI) and the amplitude (0.8684 to 0.9538 95% CI), so our procedure appeared reliable.

## 5. Conclusions

To our knowledge, this is the first approach focusing on the intrasession phase variability. The evaluation of intraindividual and intrasession variability signal has undeniable advantages in that it minimizes interindividual variations.

This research has several limitations. In our study, we limited the variables that could contribute to measurement artifacts including cataracts or other media opacities, poor visual acuity, and pupillary miosis, but the loss of fixation of the patient cannot be quantified with our technology.

The test-retest variability should vary with the dynamic range of each instrument, so each laboratory should determine its own variability on a normative sample of healthy subjects. In our study the variability of the signal does not correlate with age, but our control sample is too small to know whether any normative database should be corrected for age.

In clinical practice it is not easy to identify with certainty the actual visual acuity at a distance of work and the transparency of the dioptric media that can compromise the signal-to-noise ratio.

The values of the impedances should be constantly controlled by the operator, but most of the tools do not have these characteristics.

Standardizing the SNR is not an easy task and the assumptions of this work are governed by the reliability and experience of the laboratory, especially when working with a steady-state stimulus [25].

Further studies are needed, but our hypothesis is that increased phase variability of intrasession PERG may represent an opportunity to detect RGCs dysfunction preceding cell death.

## Figures and Tables

**Table 1:** Demographic data.

	Patients						
	EG (37)		GS (21)		NC (24)		*P* value^*^
	Mean	SD ±	Mean	SD ±	Mean	SD ±	
Age	57.1	11.6	56.1	10.5	53	6.3	*P* = 0.46
Male (%)	40.5		42.4		41.5		*P* = 0.078^*∗∗*^
IOP (mmHg)	15.6	1.1	17.9^ac^	1.6	15.1	1.7	*P* < 0.001
CCT (*μ*m)	546.6	26.6	553.1	32.1	558.7	19.1	*P* = 0.337
MD (dB)	−3.010^ab^	1.95	0.004	1.04	−0.02	1.5	*P* < 0.001
PSD (dB)	3.45^ab^	2.04	1.59	0.49	1.47	0.23	*P* < 0.001
RNFL (*μ*m)	81.56^ab^	9.26	90.8	7.5	94.9	10.1	*P* < 0.001
GCC (*μ*m)	76.13^ab^	6.88	82.09	6.53	84.7	4.9	*P* < 0.001
Amplitude (*μ*V)	0.96^ab^	0.33	0.96^a^	0.27	1.2	0.26	*P* = 0.028
Phase (*π* rad)	−0.06	0.36	−0.01	0.26	0.19	0.38	*P* = 0.069
CV_amp (%)	20.25^ab^	13.52	14.27^a^	7.05	9.42	3.99	*P* = 0.004
CV_phase (%)	8.97^b^	2.52	7.30^a^	2.51	3.4	1.13	*P* < 0.001

^*∗*^One-way analysis of variance (Bonferroni corrected).

^*∗∗*^Chi-square.

^a^Statistically significant difference from normal control (NC).

^b^Statistically significant difference from glaucoma suspects (GS).

^c^Statistically significant difference from early manifest glaucoma patients (EG).

**Table 2:** Clinical characteristics of 37 early glaucoma patients.

EGp	Age	Eye	ph1	ph2	ph3	ph4	ph5	amp1	amp2	amp3	amp4	amp5	MD	PSD	RNFL	GCC	CV_p	CV_a	Phase	Ampl	SD_p	SD_a	CCT	IOP
			(*π* rad)	(*π* rad)	(*π* rad)	(*π* rad)	(*π* rad)	(*μ*V)	(*μ*V)	(*μ*V)	(*μ*V)	(*μ*V)	(db)	(db)	(*μ*m)	(*μ*m)	(%)	(%)	(*π* rad)	(*μ*V)			(*μ*m)	(mmHg)
#1	49	R	0.09	0.40	0.16	0.33	−0.11	1.57	1.47	1.44	1.43	1.38	−3	2.84	89	83	8.32	4.18	0.17	1.46	0.18	0.06	560	16
#2	49	L	0.27	0.44	0.72	0.60	0.33	1.52	1.24	0.81	0.32	1.37	−2	7	75	70	6.67	41.5	0.47	1.05	0.17	0.44	543	15
#3	69	R	0.24	0.35	0.18	0.19	−0.01	0.73	0.60	0.63	0.75	0.57	−1.02	1.89	82	80	5.42	10.8	0.19	0.66	0.12	0.07	515	14
#4	62	R	0.12	0.42	0.52	0.63	0.63	1.61	1.87	1.70	1.86	1.80	−4	4.29	92	83	7.76	5.52	0.46	1.77	0.19	0.1	537	15
#5	46	L	−0.61	−0.43	−0.80	−0.50	−0.30	1.11	0.81	0.84	0.95	0.79	−0.74	1.25	82	78	11.3	13.5	−0.53	0.9	0.17	0.12	595	17
#6	65	R	−0.44	−0.30	−0.58	−0.42	−0.29	1.34	0.72	1.11	1.06	0.74	−5.9	7.85	74	71	6.53	23.6	−0.41	0.99	0.1	0.23	518	14
#7	60	L	−0.16	0.34	−0.03	0.09	−0.04	1.20	1.04	1.12	0.96	1.01	−2.14	4.36	63	77	8.39	7.93	0.04	1.07	0.17	0.08	540	14
#8	45	R	−0.49	−0.80	−0.55	−0.45	−0.77	0.70	0.60	0.18	0.40	0.91	−6.25	1.76	95	82	10.5	44.9	−0.61	0.56	0.15	0.25	612	17
#9	63	L	0.01	−0.44	0.20	0.32	−0.29	1.03	0.70	0.94	0.88	0.71	−3.54	2.65	100	94	14.6	15.1	−0.04	0.85	0.29	0.13	560	15
#10	35	R	0.22	−0.18	0.12	0.27	0.41	1.85	1.45	1.29	1.46	1.30	−6.11	2.18	71	77	9.06	13.7	0.17	1.47	0.2	0.2	550	16
#11	59	L	−0.26	0.32	0.47	0.06	0.35	1.11	0.67	0.78	0.76	0.79	−2.01	1.22	73	70	11.9	18.4	0.19	0.82	0.26	0.15	530	15
#12	39	L	−0.37	−0.10	−0.19	0.27	0.10	1.21	0.77	1.04	0.79	0.82	−1.5	1.87	83	77	11.5	18.3	−0.06	0.93	0.22	0.17	525	15
#13	57	R	0.13	−0.08	0.14	−0.01	0.20	1.42	1.48	0.94	1.11	1.00	−5.11	7.75	77	67	5.07	18.5	0.08	1.19	0.11	0.22	570	16
#14	54	L	−0.51	−0.31	−0.22	0.01	0.07	1.37	1.17	1.16	1.35	0.97	−3.21	2.79	97	85	11.7	12.4	−0.19	1.2	0.21	0.15	595	17
#15	59	L	0.36	0.76	0.10	0.27	0.36	1.05	0.79	1.10	1.03	1.11	−1.11	2.94	95	84	9.1	11.6	0.37	1.02	0.22	0.12	560	16
#16	75	R	−0.72	−0.08	−0.20	−0.06	−0.10	0.76	0.92	0.75	0.82	0.83	−2.13	3.55	87	88	14.1	7.46	−0.23	0.82	0.25	0.06	525	17
#17	65	R	−0.31	−0.59	−0.29	−0.31	−0.46	0.77	1.03	1.05	0.60	0.87	−3.57	2.61	92	86	7.09	19.4	−0.39	0.87	0.11	0.17	505	16
#18	60	R	0.19	0.32	−0.08	0.19	−0.19	0.56	0.52	0.87	0.56	0.62	−5.65	3.96	85	69	9.07	20	0.09	0.63	0.19	0.12	543	15
#19	81	R	−0.97	−0.63	−0.86	−0.71	−0.92	1.13	0.40	0.79	0.39	1.01	0.35	1.38	83	68	10.8	40.9	−0.82	0.75	0.13	0.3	575	16
#20	45	R	0.22	0.58	0.51	0.66	0.33	1.41	1.52	1.49	0.92	1.07	−1.2	6.3	73	69	6.58	18.9	0.46	1.28	0.16	0.24	555	15
#21	40	L	−0.11	0.11	0.32	0.18	0.55	1.82	1.58	1.06	1.85	1.46	−2.06	2.38	77	74	10	18.6	0.21	1.55	0.22	0.29	523	14
#22	72	L	0.48	0.12	0.50	0.37	0.48	1.18	0.93	1.19	0.98	0.83	−2.89	3.03	78	72	5.88	13.8	0.39	1.02	0.14	0.14	545	14
#23	52	R	−0.57	−0.38	−0.04	−0.21	−0.31	1.45	1.54	1.39	1.13	1.55	−4.5	1.62	90	82	10.3	10.8	−0.3	1.41	0.17	0.15	569	15
#24	76	L	0.12	−0.28	0.03	−0.07	−0.44	0.35	1.59	0.66	0.35	0.35	−6.49	3.76	78	66	10.9	72.7	−0.13	0.66	0.2	0.48	555	15
#25	40	R	−0.20	0.08	−0.06	0.03	0.09	1.02	0.64	0.75	0.71	0.79	−3.22	5.72	69	77	5.55	16.8	−0.01	0.78	0.11	0.13	525	16
#26	49	R	0.04	−0.04	0.07	0.27	−0.41	1.16	1.03	0.99	1.00	1.18	−2.15	1.08	77	67	11.3	7.81	−0.02	1.07	0.22	0.08	495	17
#27	43	L	−0.37	−0.19	0.05	−0.14	−0.21	0.88	1.67	1.28	1.21	0.71	−1.52	2.33	88	74	7.5	29.1	−0.17	1.15	0.14	0.33	536	15
#28	68	L	−0.09	−0.41	−0.04	−0.49	−0.07	1.18	1.38	0.88	1.22	1.04	−0.06	1.48	96	84	10.7	15	−0.22	1.14	0.19	0.17	520	16
#29	49	R	0.75	0.14	0.57	0.58	0.54	0.88	0.85	0.64	1.04	0.57	−4.55	2.24	71	74	7.99	21.2	0.52	0.8	0.2	0.17	578	17
#30	70	R	0.45	0.68	0.22	0.74	0.26	0.61	0.61	0.52	0.51	0.38	−0.45	1.59	78	76	8.64	15.5	0.47	0.53	0.21	0.08	542	17
#31	67	R	−0.43	−0.82	−0.45	−0.45	−0.29	0.40	0.34	0.29	0.40	0.77	−5.94	7.15	80	78	11.6	38.5	−0.49	0.44	0.18	0.17	523	17
#32	42	R	−0.71	−0.91	−0.86	−0.62	−0.72	0.24	0.26	0.54	0.44	0.63	−2.26	3.45	86	69	8.52	36	−0.76	0.42	0.11	0.15	555	18
#33	65	R	−0.41	−0.17	0.04	0.15	−0.05	1.64	1.17	1.02	1.31	1.02	−2.25	3.32	88	73	10.1	18.7	−0.09	1.23	0.19	0.23	567	17
#34	62	L	−0.85	−0.77	−0.82	−0.71	−0.49	0.43	0.29	0.48	0.42	0.24	−1.16	1.69	76	79	10.1	24.7	−0.73	0.37	0.13	0.09	545	17
#35	63	L	−0.26	−0.10	−0.22	0.02	−0.24	1.38	1.32	1.11	1.43	1.24	−2.06	3.92	81	72	5.7	8.65	−0.16	1.3	0.11	0.11	515	15
#36	58	R	−0.04	−0.01	0.16	0.06	−0.03	1.12	1.18	0.81	1.05	0.67	−6.71	7.78	60	76	3.74	19.9	0.03	0.97	0.08	0.19	535	15
#37	61	L	−0.33	−0.29	0.07	−0.20	−0.26	0.67	0.75	0.56	0.48	0.65	−3.25	5.02	70	76	8.09	15.3	−0.2	0.62	0.15	0.1	585	14

PERG phase and amplitude (values of 5 consecutive tests), mean deviation (MD), pattern standard deviation (PSD), retinal nerve fiber layer thickness (RNFL), ganglion cell complex (GCC), coefficient of variation of amplitude (CV_a), coefficient of variation of phase (CV_p), mean phase (phase), mean amplitude (Ampl), standard deviation of amplitude (SD_a), standard deviation of phase (CV_p), central corneal thickness (CCTi), and intraocular pressure (IOP) in 37 early glaucoma patients (EGp).

**Table 3:** Clinical characteristics of 21 glaucoma suspect patients.

SGp	Age	Eye	ph1	ph2	ph3	ph4	ph5	amp1	amp2	amp3	amp4	amp5	MD	PSD	RNFL	GCC	CV_p	CV_a	Phase	Ampl	SD_p	SD_a	CCT	IOP
			(*π* rad)	(*π* rad)	(*π* rad)	(*π* rad)	(*π* rad)	(*μ*V)	(*μ*V)	(*μ*V)	(*μ*V)	(*μ*V)	(db)	(db)	(*μ*m)	(*μ*m)	(%)	(%)	(*π* rad)	(*μ*V)			(*μ*m)	(mmHg)
#1	47	R	0.60	0.20	0.25	0.33	0.16	1.35	1.30	1.32	1.49	1.13	1.51	1.36	86	83	6.79	8.75	0.31	1.32	0.16	0.12	535	16
#2	54	R	0.04	−0.30	−0.37	−0.20	−0.08	1.00	1.01	0.88	0.83	0.76	−0.23	1.24	92	90	8.09	10.67	−0.18	0.9	0.15	0.1	570	16
#3	49	L	0.12	−0.24	−0.01	0.17	0.10	1.10	1.08	0.95	0.95	0.92	−0.59	1.78	90	78	7.23	7.69	0.03	1	0.15	0.08	597	18
#4	53	R	−0.20	0.12	0.04	−0.04	−0.08	0.95	0.73	0.87	0.89	0.70	−0.21	1.88	91	86	5.52	11.72	−0.03	1	0.11	0.1	515	18
#5	58	L	0.33	0.24	0.01	0.32	0.21	1.47	1.35	1.09	1.51	1.19	−0.78	1.88	84	79	5.11	12.02	0.22	1.32	0.11	0.16	565	20
#6	57	R	−0.16	0.17	0.27	−0.30	0.10	0.59	0.43	0.48	0.66	0.49	0.62	1.16	84	98	10.5	15.79	0.02	0.53	0.21	0.08	525	18
#7	73	L	−0.06	−0.03	−0.04	−0.08	0.17	1.43	1.05	0.87	1.08	1.23	−0.33	1.63	93	74	4.61	16.73	−0.01	1.13	0.09	0.19	549	16
#8	35	R	0.25	0.71	0.66	0.79	0.74	1.58	1.16	1.29	1.62	1.31	−0.25	1.23	96	78	7.3	12.76	0.63	1.39	0.19	0.18	508	16
#9	63	R	0.04	0.42	0.14	0.07	0.14	0.89	0.82	0.98	0.64	0.89	−0.74	1.87	108	91	6.24	13.44	0.16	1.01	0.13	0.11	578	16
#10	77	R	0.14	0.00	0.15	0.01	0.24	0.97	0.56	0.98	0.67	0.64	0.65	1.24	93	72	4.31	23.01	0.11	0.92	0.09	0.18	562	17
#11	53	L	0.42	0.33	0.11	0.37	0.38	0.94	0.65	0.69	0.54	0.66	0.02	1.33	80	80	4.72	19.1	0.32	0.7	0.11	0.13	601	18
#12	64	L	−0.04	0.00	−0.39	−0.16	−0.11	0.70	0.89	0.34	0.37	0.40	−0.01	1.23	96	88	7.25	40.32	−0.14	0.65	0.13	0.22	555	17
#13	50	L	−0.52	−0.64	−0.13	−0.34	−0.33	1.00	0.58	0.65	0.78	0.89	−0.66	1.33	87	82	10.78	19.59	−0.39	0.94	0.17	0.15	495	22
#14	62	L	−0.02	0.20	−0.03	0.23	0.14	0.53	0.40	0.51	0.59	0.48	0.68	1.1	75	74	5.25	12.25	0.1	0.5	0.11	0.06	575	18
#15	57	R	−0.51	−0.68	−0.66	−0.33	−0.62	1.34	1.54	0.86	1.03	0.65	0.84	1.24	93	86	8.93	29.8	−0.56	1.08	0.13	0.32	535	18
#16	64	L	−0.31	−0.15	−0.05	−0.07	0.20	0.66	0.67	0.73	0.55	0.72	1.43	1.4	87	73	8.52	9.33	−0.07	0.8	0.16	0.06	560	21
#17	59	L	−0.12	−0.02	0.05	−0.16	0.01	1.29	1.56	1.45	1.16	1.60	0.27	1.53	94	83	4.04	11.75	−0.05	1.41	0.08	0.17	525	18
#18	51	L	−0.41	0.00	−0.33	−0.12	−0.40	0.65	0.64	0.66	0.66	0.58	0.88	1.43	93	84	9.36	5.09	−0.25	0.77	0.16	0.03	565	18
#19	32	L	−0.09	0.18	0.12	0.03	0.21	1.40	1.42	1.12	1.17	1.24	−0.37	1.34	89	82	5.29	9.76	0.09	1.27	0.11	0.12	608	18
#20	60	L	−0.30	−0.46	−0.22	−0.52	0.13	0.78	0.70	0.66	0.47	0.85	0.25	2.08	106	85	13.18	18.58	−0.27	0.69	0.23	0.13	512	18
#21	61	R	−0.22	−0.17	0.00	−0.35	−0.54	0.82	0.64	0.63	0.88	0.88	0.99	1.45	90	78	10.38	14.46	−0.26	0.93	0.18	0.11	582	20

PERG phase and amplitude (values of 5 consecutive tests), mean deviation (MD), pattern standard deviation (PSD), retinal nerve fiber layer thickness (RNFL), ganglion cell complex (GCC), coefficient of variation of amplitude (CV_a), coefficient of variation of phase (CV_p), mean phase (phase), mean amplitude (Ampl), standard deviation of amplitude (SD_a), standard deviation of phase (CV_p), central corneal thickness (CCTi), and intraocular pressure (IOP) in 21 glaucoma suspect patients (SGp).

**Table 4:** Pearson correlation coefficient (CC) and significance level *P* (SL-*P*) in 37 early manifest glaucoma patients.

		Age	IOP	CCT	MD	PSD	RNFL	GCC	Amplitude	Phase	CV_amp	CV_phase
			(mmHg)	(*µ*m)	(dB)	(dB)	(*µ*m)	(*µ*m)	(*µ*V)	(*π* rad)	(%)	(%)
Age	CC		−0.108	−0.133	0.082	0.038	0.133	0.051	−0.181	−0.128	0.126	0.105
	SL-*P*		0.523	0.433	0.629	0.823	0.432	0.762	0.284	0.449	0.457	0.536

IOP (mmHg)	CC	−0.108		0.269	0.078	−0.252	0.296	0.177	−0.251	−0.364	0.11	0.362
	SL-*P*	0.523		0.108	0.646	0.133	0.075	0.294	0.134	0.026	0.516	0.0277

CCT (*μ*m)	CC	−0.133	0.269		−0.131	−0.108	0.181	0.019	0.043	−0.157	0.207	0.178
	SL-*P*	0.433	0.108		0.441	0.526	0.282	0.908	0.8	0.352	0.218	0.292

MD (dB)	CC	0.082	0.078	−0.131		−0.465	0.117	0.05	0.025	0.011	−0.287	0.153
	SL-*P*	0.629	0.646	0.441		0.0037	0.491	0.766	0.882	0.949	0.084	0.366

PSD (dB)	CC	0.038	−0.252	−0.108	−0.465		−0.386	−0.279	0.024	0.138	0.152	−0.524
	SL-*P*	0.823	0.133	0.526	0.0037		0.018	0.0945	0.889	0.414	0.369	0.0009

RNFL (*μ*m)	CC	0.133	0.296	0.181	0.117	−0.386		0.636	0.444	−0.228	−0.05	0.427
	SL-*P*	0.432	0.075	0.282	0.491	0.0184		<0.0001	0.005	0.175	0.768	0.0083

GCC (*μ*m)	CC	0.051	0.177	0.019	0.05	−0.279	0.636		0.24	−0.054	−0.379	0.361
	SL-*P*	0.762	0.294	0.908	0.766	0.094	<0.0001		0.152	0.753	0.0206	0.0283

Amplitude (*μ*V)	CC	−0.181	−0.251	0.043	0.025	0.024	0.444	0.24		0.285	−0.32	−0.037
	SL-*P*	0.284	0.134	0.8	0.882	0.889	0.0059	0.152		0.087	0.0536	0.828

Phase (*π* rad)	CC	−0.128	−0.364	−0.157	0.011	0.138	−0.228	−0.054	0.285		−0.346	−0.33
	SL-*P*	0.449	0.0268	0.352	0.949	0.414	0.175	0.753	0.087		0.0358	0.0461

CV_amp (%)	CC	0.126	0.11	0.207	−0.287	0.152	−0.05	−0.379	−0.32	−0.346		0.09
	SL-*P*	0.457	0.516	0.218	0.084	0.369	0.768	*0.02 *	0.053	0.0358		0.597

CV_phase (%)	CC	0.105	0.362	0.178	0.153	−0.524	0.427	0.361	−0.037	−0.33	0.09	
	SL-*P*	0.536	0.0277	0.292	0.368	0.0009	0.0083	0.028	0.828	0.0461	0.597	

Intraocular pressure (IOP), central corneal thickness (CCT), mean deviation (MD), pattern standard deviation (PSD), retinal nerve fiber layer thickness (RNFL), ganglion cell complex (GCC), PERG amplitude, PERG phase, coefficient of variation PERG amplitude (CV_amp), and coefficient of variation PERG phase (CV_Phase).

**Table 5:** Intraclass correlation coefficient.

	Patients					
	EG (37)		GS (21)		NC (24)	
	ICC^a^	95% CI	ICC^a^	95% CI	ICC^a^	95% CI
PERG amplitude (*μ*V)	0.9187	0.8684 to 0.9538	0.9466	0.8993 to 0.9756	0.9642	0.9226 to 0.9869
PERG phase (*π* rad)	0.9459	0.9124 to 0.9692	0.9232	0.8554 to 0.9650	0.9879	0.9739 to 0.9956

^a^The degree of consistency among measurements.

ICC = intraclass correlation coefficient.

IC = confidence interval.
